# Dynamic control of Purcell enhanced emission of erbium ions in nanoparticles

**DOI:** 10.1038/s41467-021-23632-9

**Published:** 2021-06-11

**Authors:** Bernardo Casabone, Chetan Deshmukh, Shuping Liu, Diana Serrano, Alban Ferrier, Thomas Hümmer, Philippe Goldner, David Hunger, Hugues de Riedmatten

**Affiliations:** 1 ICFO-Institut de Ciencies Fotoniques, The Barcelona Institute of Science and Technology, Barcelona, Spain; 2 Chimie ParisTech, PSL University, CNRS, Institut de Recherche de Chimie Paris, Paris, France; 3 Shenzhen Institute for Quantum Science and Engineering, Southern University of Science and Technology, Shenzhen, China; 4 Faculté des Sciences et Ingénierie, Sorbonne Université, Paris, France; 5 Fakultät für Physik, Ludwig-Maximilians-Universität, München, Germany; 6 Karlsruher Institut für Technologie, Physikalisches Institut, Karlsruhe, Germany; 7 Karlsruhe Insitute for Technology, Institute for Quantum Materials and Technologies (IQMT), Eggenstein-Leopoldshafen, Germany; 8 ICREA-Institució Catalana de Recerca i Estudis Avançats, Barcelona, Spain

**Keywords:** Quantum optics, Quantum optics, Quantum information

## Abstract

The interaction of single quantum emitters with an optical cavity enables the realization of efficient spin-photon interfaces, an essential resource for quantum networks. The dynamical control of the spontaneous emission rate of quantum emitters in cavities has important implications in quantum technologies, e.g., for shaping the emitted photons’ waveform or for driving coherently the optical transition while preventing photon emission. Here we demonstrate the dynamical control of the Purcell enhanced emission of a small ensemble of erbium ions doped into a nanoparticle. By embedding the nanoparticles into a fully tunable high finesse fiber based optical microcavity, we demonstrate a median Purcell factor of 15 for the ensemble of ions. We also show that we can dynamically control the Purcell enhanced emission by tuning the cavity on and out of resonance, by controlling its length with sub-nanometer precision on a time scale more than two orders of magnitude faster than the natural lifetime of the erbium ions. This capability opens prospects for the realization of efficient nanoscale quantum interfaces between solid-state spins and single telecom photons with controllable waveform, for non-destructive detection of photonic qubits, and for the realization of quantum gates between rare-earth ion qubits coupled to an optical cavity.

## Abstract (web summary)

Control of quantum emitters is needed in order to enable many applications. Here, the authors demonstrate enhancement and dynamical control of the Purcell emission from erbium ions doped in a nanoparticle within a fiber-based microcavity.

## Introduction

Quantum network nodes should be able to store quantum information for long durations, to process it using local quantum gates between qubits, and to exchange this information with distant nodes, ideally using photons at telecommunication wavelengths, via an efficient spin–photon interface^1^. Several platforms are currently investigated for the realization of quantum nodes, including atoms, trapped ions, and solid-state systems^2,3^. Single atoms or solid-state emitters provide a platform for spin–photon interfaces with quantum processing capabilities^3,4^. The realization of an efficient spin–photon interface is facilitated by the use of an optical cavity in the Purcell regime^5–8^, which allows channeling the emission from the emitter in the cavity mode while decreasing the spontaneous emission lifetime. In the presence of dephasing, it also increases the indistinguishability of photons from the emitter. However, a strong reduction of the optical lifetime also reduces the available time to realize quantum gates that rely on dipole-blockade mechanisms achieved by driving the emitter to the excited state^9^. One attractive solution to this problem is to decouple the emitters from the cavity when performing the quantum gates, and coupling it back to emit a single photon at a desired time. The ability to achieve a dynamic modulation of the Purcell factor would therefore be a key ingredient to achieve this type of quantum gates in a high-efficiency spin–photon interface, as well as an essential tool to shape the emitted single-photon waveform and to realize cavity-enhanced non-destructive detection of photonic qubits^10^. In addition, the dynamic resonance modulation also enables addressing inhomogeneously broadened ensembles of single emitters as multi-qubit registers.

The control of collective light emission from ensembles of atoms in free space has been demonstrated^11,12^. Experiments toward the dynamic control of emission of the cavity-enhanced spontaneous emission rate have been so far mostly performed with semi-conductor quantum dots featuring short optical and spin coherence time^13–16^, with Raman schemes with single atoms^17^ and ions^18^, and by modifying the local optical environment of rare-earth ion-doped (REI) crystals^19,20^. Related work on fast modulation of erbium ions emission has been performed recently, using electrical modulation of erbium–graphene interaction^21^. Among solid-state materials, REI crystals constitute a promising platform for quantum information processing and networking. REI feature exceptional spin coherence time^22–24^ to store information and narrow optical transitions as spin–photon interface, including at telecom wavelength for erbium ions. They have been used extensively as ensemble-based quantum memories^25–29^. REI possess permanent dipole moments with different values in the ground and excited states, which enables strong dipolar interactions between nearby ions, opening the door to the realization of quantum gates between two or more matter qubits, using a dipole-blockade mechanism achieved by exciting one ion^9^, e.g., between erbium and another ion species in the context of quantum repeaters^30^.

Recently, rare-earth ions have proven to preserve their coherence properties in nanoparticles^31–33^. This facilitates their incorporation into microcavities to reach strong Purcell enhancement, as necessary for the emission of coherent indistinguishable single photons. Also, it provides the necessary confinement required to isolate close-by single ion qubits (≈10-nm distance), as required for dipolar quantum gates. Following the first demonstration in free space^34–36^, REIs coupled to nanophotonic cavities have also recently led to the detection and manipulation of single rare-earth ions^37–40^.

In this work, we demonstrate the dynamical control of the Purcell enhanced emission of a small ensemble of erbium ions in a single nanoparticle. This is achieved by inserting the nanopaticles into a fully tunable high finesse fiber microcavity at cryogenic temperature, whose frequency can be tuned at rates more than 100 times faster than the spontaneous emission lifetime of 10.8(3) ms of the ions by physically moving the fiber mirror with sub-nanometer precision using a piezoelectric device. By measuring the decay of fluorescence counts in the cavity, we infer an average effective Purcell enhancement of 14(1). The decay is compatible with a multiexponential model describing Purcell enhancement with an ensemble of ions that predicts that 10% of the ions experience an effective Purcell enhancement higher than 74. The Purcell enhancement can be controlled on a time scale of hundred microseconds. In addition, we show that we can shape in real time the Purcell enhanced emission of the ions to control the emitted photons’ waveforms, without perturbing the emitter. Our approach opens the door to a solid-state quantum node with the potential of exhibiting quantum computing and communication capabilities all in a single device.

## Results

### Description of the experiment

When coupling an emitter to a microcavity the emitter’s lifetime is reduced to *τ*_*c*_ = *τ*_*n*_/(*C* + 1), where *τ*_*n*_ is the natural lifetime and $C={\mathcal{L}}\zeta {C}_{0}={\mathcal{L}}\zeta \frac{3}{4{\pi }^{2}}\frac{Q}{{V}_{m}}{\lambda }^{3}$ is the effective Purcell factor, with *λ* the emission wavelength, *Q* the quality factor of the resonator and *V*_*m*_ its mode volume, *ζ* the branching ratio of the respective transition and ${\mathcal{L(\delta )}}=\frac{{({{\Delta }}/2)}^{2}}{{\delta }^{2}+{({{\Delta }}/2)}^{2}}$ is the detuning factor in case the cavity with linewidth Δ is detuned by *δ* from the emitter’s resonance. In addition, the collection efficiency of the cavity mode is given by *β* = *ηC*/(*C* + 1) where *η* is the cavity outcoupling efficiency. Thus, fast and near-unity efficiency readout can be achieved for sufficiently large *C* and *η*.

Fiber cavities can achieve high Purcell factors up to 10^4 ^^41^, provide open access to the cavity mode for optimal overlap between the ions and the cavity field^42–44^ and are small and lightweight enough to offer fast frequency tuning. Nanoparticles are a promising platform to study REI as their small size isolates a mesoscopic ion number, such that the large ratio between the inhomogeneous and homogeneous linewidth can allow one to frequency select single ions^37,38^. Also, their scattering cross section can be small enough such that integration into high-finesse cavities is possible. In particular, we study Er^3+^:Y_2_O_3_ nanoparticles with 200 ppm erbium concentration and an average radius of 75 nm. Y_2_O_3_ is a host that showed to maintain good crystalline quality in the nanoscale allowing long coherence time for dopants like europium^31,32^ and praseodymium^33^. For Er^3+^:Y_2_O_3_, the optical transition ^4^*I*_15/2_–^4^*I*_13/2_ is at 1535 nm with a relatively large branching ratio *ζ* = 0.21.

**Fig. 1:**
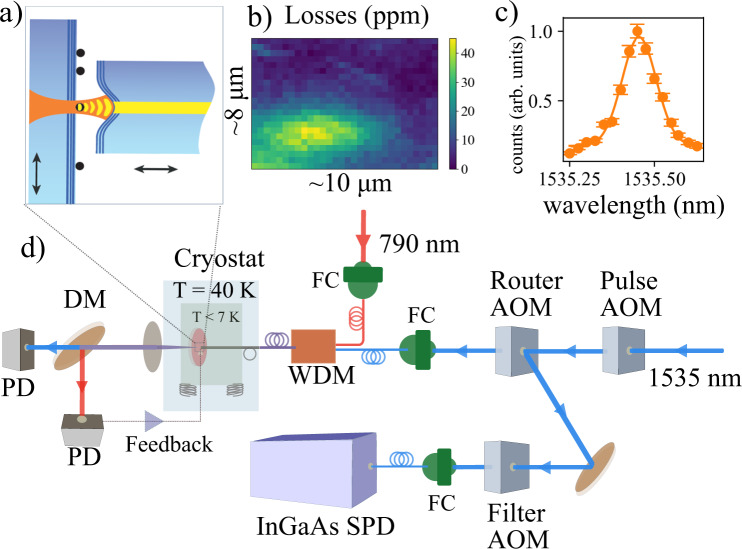
Description of the experiment. **a** Schematic drawing of the tunable fiber-based microcavity. The fiber mirror can be moved in 3D, allowing us to set the length of the cavity and locate a nanoparticle inside the cavity. **b** Map of scattering loss introduced by a single nanoparticle when scanning the cavity mode across it. **c** Measurement of the inhomogeneous line of the ^4^*I*_15/2_–^4^*I*_13/2_ transition. The solid line is a Gaussian fit, yielding a full-width-half-maximum linewidth of 15(2) GHz (error bars represent one standard deviation). **d** The optical setup is described in more detail in the SI. In summary, a 1535-nm laser is used to excite the ions and a 790-nm laser to stabilize the length of the cavity. The two lasers are combined using a wavelength division multiplexer (WDM). A set of acousto-optic modulators (AOM) are used to create pulses from the CW excitation laser, to route the excitation light to the cavity and the cavity photons to the detector, and to suppress the excitation light to the single-photon detector (SPD) by 60 dB (InGaAs, detection efficiency 10%) during excitation. DM is a 780/1535 nm dichroic mirror, PD are continuous avalanche photo-diodes (APD) for cavity length stabilization and transmission monitoring.

Our setup is based on a fast and fully tunable, stable, cryogenic-compatible Fabry–Perot microcavity (see Fig. 1a). The microcavity is composed of a fiber with a concave structure on the tip, on which a reflective coating is deposited. The other side of the cavity is a planar mirror with the same reflective coating as the fiber, over which the doped nanoparticles are placed after depositing a thin layer of SiO_2_ to ensure maximum coupling between the ions and the cavity electric field (see section 1 of Supplementary Information (SI)). The setup allows to move the fiber around the mirror and localize the nanoparticles, and to set the separation between the fiber and the mirror to form a cavity on resonance with the ions. Nanoparticles are located by scattering loss microscopy^44–46^. We use a nanoparticle with a radius of 91(1) nm (see Fig. 1b) adding a total intra-cavity loss of 2 × 43(2) parts-per-million (ppm) per round trip and containing close to 11,000 Er^3+^ ions in the measured *C*_2_ crystallographic site. The empty cavity has a finesse of 2 × 10^4^, which is reduced to 1.6 × 10^4^ in presence of the nanoparticle. For cavities as short as 3.5 μm we can expect a maximal Purcell factor ${C}_{\max }=320$, which then reduces to 176 for a cavity length of 6 μm as used in the work presented here.

To ensure the highest Purcell factor, a cavity length stability smaller than $\frac{\lambda }{2F}\approx 40$ pm is required. We have built a compact and passively stable nano-positioning platform which is robust against the high-frequency noise coming from the closed-cycle cryostat such that active stabilization in the low-frequency domain, i.e., below 1 kHz, is enough to stabilize the cavity (see section 3 of SI). We use a second laser at 790 nm to actively stabilize the length of the cavity on an arbitrary point on the transmission fringe. The coating is designed to have a finesse of 2000 around 790 nm and such that there is a red mode close by to all 1535 nm resonances for cavity lengths in the 2–20 μm range.

We were able to stabilize the cavity length to a chosen longitudinal mode with a root mean square (RMS) stability of 31 pm during the whole cycle of the cryostat. For the measurements presented here, the stability, however, is lower and typical values between 50 and 75 pm RMS were recorded (see section 3 of SI). The temperature of the sample-mirror was below 7 K.

In order to perform experiments, we use resonant excitation and detection both via the optical fiber (see Fig. 1c). The probability for a photon emitted in the cavity mode to reach the single-photon detector is 2.8% and the coupling efficiency between the fiber and the cavity mode is calculated to be 55% (see section 7 of SI).

### Measurements of Purcell enhancement

We first perform resonant cavity spectroscopy of the ^4^*I*_15/2_–^4^*I*_13/2_ transition with an input power (in the input fiber) of 2.4 μW. We excite the ions for 300 μs, then wait for 50 μs to ensure the pulse AOM is completely switched off, and collect light for 5 ms. Figure 1c shows the normalized counts in the detection window as a function of the excitation wavelength. We fit it with a Gaussian profile and extract a FWHM linewidth of 15(2) GHz centered at 1535.42 nm.

**Fig. 2:**
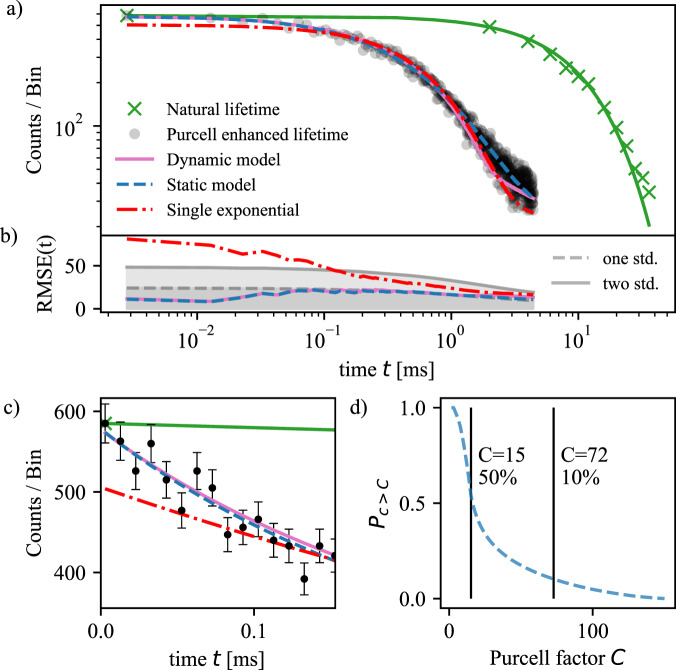
Purcell enhanced lifetime measurements. **a** Black points and green crosses correspond to the natural and Purcell enhanced lifetime measurements. Dash-dotted red line: Single exponential fit. Solid magenta and dashed blue line are the dynamic and static model, respectively. **b** Gray lines correspond to one and two standard deviation calculated by summing all the counts in the detection window [*t*_0_, *t*] of the Purcell enhanced lifetime measurement (black points), where *t*_0_ is the time at which the photon counting is started, and considering Poissonian counting statistics. Root-mean-square error (RMSE) of the single exponential, the static, and the dynamic models, also calculated as function of the detection time window. The single exponential fit deviates by more than two standard deviations at earlier times, while the model and the reconstructed decaying curve stay below this threshold. **c** Zoom of the first 150 μs of the plot shown in (**a**). Error bars represent one standard deviation calculated from Poisson counting statistics. **d** Estimated probability *P*_*c*>*C*_ that a given ion decays with a Purcell factor *c* *>* *C*.

We then fix the frequency of the laser to be in the center of the line and perform lifetime measurements (input power of 11 μW). Figure 2a shows two data sets. Black points and green crosses correspond to the Purcell enhanced and natural lifetime measurements, respectively. Single exponential fits reveal Purcell enhanced and natural lifetimes *τ*_*c*_ = 0.74(1) ms and *τ*_*n*_ = 10.8(3) ms (see below for a description of the procedure to extract the natural lifetime). We calculate the effective Purcell factor *C* = *C*_0_*ζ* = *τ*_*n*_/*τ*_*c*_ − 1 = 14(1) which yields *C*_0_ = 65(2) with *ζ* = 0.21 the branching ratio of the transition. We then estimate the probability for an ion to emit in the cavity mode *β* = *C*/(*C* + 1) = 93.2%, and the cavity-enhanced branching ratio *ζ*_*c*_ = *ζ*(*C*_0_ + 1)/(*ζC*_0_ + 1) = 95.5%.

The single exponential fit does not describe well the data for the Purcell enhanced case suggesting that emitters experiencing different enhancement contribute to the detected signal (see dash-dotted red line in Fig. 2a–c). To investigate this further, we derive a model describing our experiment (see section 6 of SI). The model considers a maximum Purcell factor ${C}_{\max }$ which is then reduced due to the randomly oriented dipole moment of the emitters, the finite extension of the particle^43^, and fluctuations of the cavity resonance. From the model we can perform a dynamic and a static analysis. The dynamic approach is based on the probability of an ion to emit into the cavity mode which is proportional to ${e}^{{-\int_{0}^{t}\frac{{C(t^{\prime})}}{\tau_{n}} dt^{\prime}}}$ where $C(t^{\prime} )$ is the experienced Purcell factor as function of time. The static approach starts by estimating the distribution of Purcell factors in the system $p(s=C/{C}_{\max })$, from which we reconstruct a decaying curve proportional to $\mathop{\int}\nolimits_{0}^{1}p(s){e}^{-st{C}_{\max }/{\tau }_{n}}ds$. While the first method is a direct simulation of the experiment, which is sensitive to the frequency of the cavity vibration, from the second method we can obtain a distribution of Purcell factors and estimate the maximum enhancement present in the system (see section 6 of SI).

The magenta and blue lines in Fig. 2a–c are the models following the dynamic and static analysis, respectively, when considering expected parameters from our system but reducing ${C}_{\max }$ to 150 in order to obtain a satisfying description of the experimental data. Figure 2d is the probability *P*_*c*>*C*_ that a given ion experiences a Purcell factor *c* greater than *C* calculated from the estimated *p*(*s*). From this model, we infer that 50% of the ions experience a Purcell factor larger than *C* = 15 consistent with the measured *τ*_*c*_ = 0.74(1) ms, and that at least 10% of the ions experience a Purcell factor larger than 74. The models describe the data with noticeably more accuracy than the single exponential (see RMSE calculations in Fig. 2b). In particular, they succeed to reproduce the decay at early times, confirming the presence of Purcell enhancement larger than 15.

Black points in Fig. 2a corresponds to photons generated by a few hundred ions due to power broadening, and by decreasing the input power to 300 pW, a number as low as 10 ions could be detected with a signal-to-noise ratio around 5 (see section 7 of SI). High-fidelity single ion detection could be achieved by using more efficient superconducting nanowire single-photon detectors.

### Dynamical control of Purcell enhanced emission

**Fig. 3:**
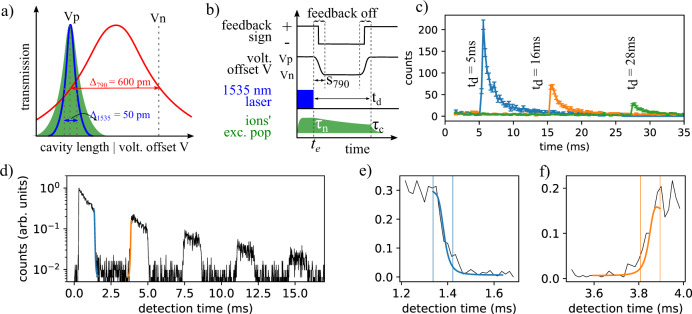
Control of Purcell enhanced emission. **a** Scheme to stabilize the length of the cavity on/off-resonance with the ions. The cavity length is controlled by applying a voltage offset *V* that moves the fiber. For *V*_*p*_, the transmission of the 790 nm locking laser is at the 50% level on the positive slope of the fringe (red curve), and on resonance with both the center of the inhomogeneous line of the ions (green area) and the 1535 nm excitation laser (blue fringe). For *V*_*n*_, the fiber is displaced by *d*_*f*_ = Δ_790_ = 600 pm with respect to the position of *V*_*p*_. The transmission of the 790 nm locking laser is at the 50% level but on the negative slope, and both the excitation 1535 nm laser and the center of the homogeneous line are detuned by 12Δ_1535_ (see main text for details). The cavity length can be stabilized to the 50% level of either 790-nm fringe, for which the feedback sign has to be adequately set. **b** Sequence used to extract *τ*_*n*_. First, *V*_*p*_ is applied for a time *t*_*e*_ and the 1535 nm resonance is driven. At a time *t*_*e*_, the voltage is switched from *V*_*p*_ to *V*_*n*_ in a time *S*_790_. The excited state population then decays at *τ*_*n*_. After a time *t*_*e*_ + *t*_*d*_ + *S*_790_, *V*_*n*_ is switched back to *V*_*p*_. Feedback sign is set to positive (negative) for *V*_*p *_(*V*_*n*_), and feedback action is off in the transient stage of the voltage. **c** Counts recorded as function of time *t* for *t*_*d*_ = 5, 16, and 28 ms. Error bars represent one standard deviation of photon counts. **d** Counts as function of time when the voltage is alternated between *V*_*p*_ and *V*_*n*_ five times at intervals of 1 ms after excitation of the ions. Zooms of the falling (**e**) and rising (**f**) edges are also shown, revealing an average transition time of *S*_1535_ = 85(15) μs for the falling edge and 87(16) μs for the rising edge (see “Discussion” in the main text).

We now explain our strategy to tune the cavity resonance in a time scale faster than the spontaneous lifetime. First, we set the 1535 nm excitation laser on resonance with the center of the inhomogeneous line of the ions. Then, we use a second laser at 790 nm to stabilize the length of the cavity. The 790-nm wavelength is tuned such that 50% transmission level of the positive slope overlaps with the center of the 1535 nm cavity resonance (see Fig. 3a). By a fast change of the voltage offset *V* from *V*_*p*_ to *V*_*n*_ or vice-versa, and by switching the sign of the feedback action, we can stabilize the cavity to the positive and negative sides of the 790-nm fringe at will (the feedback is off during the transient phase). The voltage is varied as ${\sin }^{2}(\frac{\pi t}{2{S}_{790}})$ and the process happens during a time *S*_790_ = 300 μs for our realization. Between the two locking positions, the total fiber displacement *d*_*f*_ corresponds to the full-width at half-maximum of the 790 nm cavity fringe, that is, ${d}_{f}={{{\Delta }}}_{790}=\frac{790\,{\rm{nm}}}{2{F}_{790}}\approx 600$ pm (*F*_790_ ≈ 700, reduced due to the particle losses). This displacement is 12 times larger than Δ_1535_ ≈ 50 pm, such that the detuning between the erbium ions and the 1535-nm cavity is *δ* = 12Δ_1535_. Using the Lorentzian lineshape of the cavity, one can estimate a maximum reduction of Purcell factor $C\propto {\mathcal{L(\delta )}}\approx 1/577$ and for the fluorescence count rate of 1/630^47^ (see section 4 of SI).

Next, we show the implementation of this technique and how it can be used to extract the natural lifetime *τ*_*n*_ of a single nanoparticle while isolating the effect of the cavity from any other lifetime reduction process. We first set the cavity on resonance with the ions (*V* = *V*_*p*_) and turn on the excitation laser at 1535 nm for *t*_*e*_ = 300 μs (Fig. 3b). Immediately after, we detune the cavity (*V* = *V*_*n*_) and start to collect photons. After a time *t*_*d*_, we set the cavity back on resonance (*V* = *V*_*p*_). Figure 3c shows the counts as function of time for *t*_*d*_ = 5, 16, and 28 ms. As shown in Fig. 3c, the total counts decrease while increasing *t*_*d*_. When the cavity is off-resonance with ions, the excited state population decays with the natural lifetime *τ*_*n*_, and therefore less ions contribute to the detected signal for longer *t*_*d*_. In order to extract the natural lifetime, we repeat the measurements for *t*_*d*_ in a range from 1 to 28 ms and calculate the detected number of photons in the [*t*_*d*_ − *t*_*d*_ + 5] ms time window (crosses in Fig. 2). A fit to a single exponential gives a lifetime of *τ* = 10.8(3) ms. We note that under certain conditions the off-resonant cavity is expected to lead to a suppression of spontaneous emission. Comparing our geometry to the one in ref. ^48^ and experiments with planar Fabry–Perot microcavities^49^, we estimate that the effect is small and should lead to <3% change in the lifetime. In addition, the presence of the planar mirror increases the spontaneous emission rate, and FDTD simulations predict enhancement by up to a factor 1.3, value which is consistent with measurements (see section 5 of SI). The overall Purcell factor comparing the lifetime for a resonant cavity with a nanoparticle in free space would be larger by this factor^44^.

Finally, we demonstrate that this technique can be operated with a bandwidth high enough to shape the spontaneous emission of the erbium ions. Figure 3d shows the recorded counts as function of the detection time while the cavity is tuned on (off) resonance for 1 ms (2.5 ms) five times. Figure 3e, f shows a zoom-in of the falling and rising edges. The solid lines are a model using the piezo displacement, *S*_790_, and an effective 1535-nm cavity linewidth to account for cavity instability. Defining the switching time *S*_1535_ as the time needed to decrease the count rate by a factor of 10, we extract from the model *S*_1535_ = 85(15) μs (see section 4 of SI). This value is more than two orders of magnitude shorter than the natural population decay time, and a factor of 9 shorter than the Purcell enhanced decay time *τ*_*c*_ of the ionic ensemble. For future experiments with single erbium ions (or with other ions with shorter lifetime), much higher Purcell factors and consequently much shorter *τ*_*c*_ will be required. Therefore, much shorter switching times are desirable. Several improvements in our experiment are possible to decrease *S*_1535_. First, the current measured value is slower than the expected time of 67 μs, which can be explained by the limited cavity stability (see section 4 of SI). Improvements on the cavity stability will therefore directly impact *S*_1535_. Another possibility is to increase the finesse of the cavity at 1535 nm (and therefore decrease Δ_1535_), as also required for increasing the Purcell factor. Finally, the switching time *S*_790_ could be decreased by designing a system with higher mechanical eigen frequencies or by iterative learning algorithms to minimize added noise. Altogether, we estimate that values of *S*_1535_ of a few microseconds could be achievable by combining these improvements (see section 4 of SI).

## Discussion

In conclusion, we have demonstrated the dynamical control of the Purcell enhanced emission of a mesoscopic ensemble of erbium ions confined in a nanoparticle embedded in an open fiber-based microcavity. By controlling the position of the fiber mirror, we have shown that we can control the cavity resonance and therefore the Purcell factor at a rate more than 100 times faster than the natural decay rate of the ions with the potential of reaching microseconds switching times. By varying the speed at which the fiber is displaced during the switching process, our setup would allow arbitrary shaping of the temporal waveform of the emitted photons. Combined with single-ion addressing, this ability will enable the generation of fully tunable narrowband single photons, and provides a route to quantum processing using single rare-earth ions.

While this paper was under review, we became aware of a related work showing Purcell enhanced emission of erbium dopants on a 19 μm thin film coupled to an optical cavity^50^.

## Supplementary information

Supplementary Information

## Data Availability

The data that support the findings of this study are available at Zenodo with the identifier 10.5281/zenodo.3736425.

## References

[1] Kimble HJ (2008). The quantum internet. Nature.

[2] Sangouard N, Simon C, de Riedmatten H, Gisin N (2011). Quantum repeaters based on atomic ensembles and linear optics. Rev. Mod. Phys..

[3] Northup TE, Blatt R (2014). Quantum information transfer using photons. Nat. Photon..

[4] Reiserer A, Rempe G (2015). Cavity-based quantum networks with single atoms and optical photons. Rev. Mod. Phys..

[5] Ritter S (2012). An elementary quantum network of single atoms in optical cavities. Nature.

[6] Najer D (2019). A gated quantum dot strongly coupled to an optical microcavity. Nature.

[7] Wang D (2019). Turning a molecule into a coherent two-level quantum system. Nat. Phys..

[8] Nguyen C (2019). Quantum network nodes based on diamond qubits with an efficient nanophotonic interface. Phys. Rev. Lett..

[9] Wesenberg JH, Mølmer K, Rippe L, Kröll S (2007). Scalable designs for quantum computing with rare-earth-ion-doped crystals. Phys. Rev. A.

[10] Goswami S, Heshami K, Simon C (2018). Theory of cavity-enhanced nondestructive detection of photonic qubits in a solid-state atomic ensemble. Phys. Rev. A.

[11] Minář J (2009). Electric control of collective atomic coherence in an erbium-doped solid. New J, Phys,.

[12] Farrera P (2016). Generation of single photons with highly tunable wave shape from a cold atomic ensemble. Nat, Commun,.

[13] Pagliano F (2014). Dynamically controlling the emission of single excitons in photonic crystal cavities. Nat. Commun..

[14] Bose R, Cai T, Choudhury KR, Solomon GS, Waks E (2014). All-optical coherent control of vacuum Rabi oscillations. Nat. Photon..

[15] Jin C-Y (2014). Ultrafast non-local control of spontaneous emission. Nat. Nanotechnol..

[16] Peinke, E. et al. Generation of ultrashort (10 ps) spontaneous emission pulses by quantum dots in a switched optical microcavity. Preprint at https://arxiv.org/abs/1910.10518 (2019).

[17] Morin O, Körber M, Langenfeld S, Rempe G (2019). Deterministic shaping and reshaping of single-photon temporal wave functions. Phys. Rev. Lett..

[18] Stute A (2012). Tunable ion-photon entanglement in an optical cavity. Nature.

[19] Karaveli S, Weinstein AJ, Zia R (2013). Direct modulation of lanthanide emission at sub-lifetime scales. Nano Lett..

[20] Cueff S (2015). Dynamic control of light emission faster than the lifetime limit using VO_2_ phase-change. Nat. Commun..

[21] Cano D (2020). Fast electrical modulation of strong near-field interactions between erbium emitters and graphene. Nat. Commun..

[22] Heinze G, Hubrich C, Halfmann T (2013). Stopped light and image storage by electromagnetically induced transparency up to the regime of one minute. Phys. Rev. Lett..

[23] Zhong M (2015). Optically addressable nuclear spins in a solid with a six-hour coherence time. Nature.

[24] Rančić M, Hedges MP, Ahlefeldt RL, Sellars MJ (2018). Coherence time of over a second in a telecom-compatible quantum memory storage material. Nat. Phys..

[25] de Riedmatten H, Afzelius M, Staudt MU, Simon C, Gisin N (2008). A solid-state light-matter interface at the single-photon level. Nature.

[26] Hedges MP, Longdell JJ, Li Y, Sellars MJ (2010). Efficient quantum memory for light. Nature.

[27] Clausen C (2011). Quantum storage of photonic entanglement in a crystal. Nature.

[28] Saglamyurek E (2011). Broadband waveguide quantum memory for entangled photons. Nature.

[29] Seri A (2017). Quantum correlations between single telecom photons and a multimode on-demand solid-state quantum memory. Phys. Rev. X.

[30] Kimiaee Asadi F (2018). Quantum repeaters with individual rare-earth ions at telecommunication wavelengths. Quantum.

[31] Bartholomew JG, de Oliveira Lima K, Ferrier A, Goldner P (2017). Optical line width broadening mechanisms at the 10 kHz level in Eu^3+^:Y_2_O_3_ nanoparticles. Nano Lett..

[32] Serrano D, Karlsson J, Fossati A, Ferrier A, Goldner P (2018). All-optical control of long-lived nuclear spins in rare-earth doped nanoparticles. Nat. Commun..

[33] Serrano D (2019). Coherent optical and spin spectroscopy of nanoscale Pr^3+^:Y_2_O_3_. Phys. Rev. B.

[34] Kolesov R (2012). Optical detection of a single rare-earth ion in a crystal. Nat. Commun..

[35] Kolesov R (2013). Mapping spin coherence of a single rare-earth ion in a crystal onto a single photon polarization state. Phys. Rev. Lett..

[36] Utikal T (2014). Spectroscopic detection and state preparation of a single praseodymium ion in a crystal. Nat. Commun..

[37] Dibos AM, Raha M, Phenicie CM, Thompson JD (2018). Atomic source of single photons in the telecom band. Phys. Rev. Lett..

[38] Zhong T (2018). Optically addressing single rare-earth ions in a nanophotonic cavity. Phys. Rev. Lett..

[39] Raha, M. et al. Optical quantum nondemolition measurement of a solid-state spin without a cycling transition. *Nat. Commun*. **11**, 1605 (2020). 10.1038/s41467-020-15138-7 PMC7105499 32231204

[40] Kindem, J. M. et al. Coherent control and single-shot readout of a rare-earth ion embedded in a nanophotonic cavity. *Nature* **580**, 201–204 (2020). 10.1038/s41586-020-2160-9 32269343

[41] Hunger D (2010). A fiber Fabry-Perot cavity with high finesse. New J. Phys..

[42] Albrecht R, Bommer A, Deutsch C, Reichel J, Becher C (2013). Coupling of a single nitrogen-vacancy center in diamond to a fiber-based microcavity. Phys. Rev. Lett..

[43] Benedikter J (2017). Cavity-enhanced single-photon source based on the silicon-vacancy center in diamond. Phys. Rev. Appl..

[44] Casabone B (2018). Cavity-enhanced spectroscopy of a few-ion ensemble in Eu^3+^:Y_2_O_3_. New J. Phys..

[45] Mader M, Reichel J, Hänsch TW, Hunger D (2015). A scanning cavity microscope. Nat. Commun..

[46] Wind M, Vlieger J, Bedeaux D (1987). The polarizability of a truncated sphere on a substrate I. Physica A Stat. Mech. Appl..

[47] Thyrrestrup H, Hartsuiker A, Gérard J-M, Vos WL (2013). Non-exponential spontaneous emission dynamics for emitters in a time-dependent optical cavity. Opt. Express.

[48] Di Z (2012). Controlling the emission from semiconductor quantum dots using ultra-small tunable optical microcavities. New J. Phys..

[49] Kaupp H (2016). Purcell-enhanced single-photon emission from nitrogen-vacancy centers coupled to a tunable microcavity. Phys. Rev. Appl..

[50] Merkel, B., Ulanowski, A. & Reiserer, A. Coherent emission of erbium dopants in a high-Q resonator. *Phys. Rev. X* **10**, 041025 (2020).

