# Supplementary material for: Dynamic control of Purcell enhanced emission of erbium ions in nanoparticles
Source: Nat Commun. 2021 Jun 11;12:3570. doi: 10.1038/s41467-021-23632-9 (PMC8196009; doi:10.1038/s41467-021-23632-9)
Supplement: Supplementary file 1 — Supplementary Information [file 41467_2021_23632_MOESM1_ESM.pdf]

Supplementary material: Dynamic control of Purcell enhanced emission of Er ions in nanoparticles

Bernardo Casabone,[1,10] Chetan Deshmukh,[1,10] Shuping Liu,[2,3] Diana Serrano,[2] Alban Ferrier,[2,4] Thomas Hümmer,[5] Philippe Goldner,[2] David Hunger,[6,7] and Hugues de Riedmatten[1,8,9]

[1] *ICFO-Institut de Ciencies Fotoniques, The Barcelona Institute of Science and Technology, 08860 Castelldefels, Barcelona, Spain*
[2] *Chimie ParisTech, PSL University, CNRS, Institut de Recherche de Chimie Paris, 75005 Paris, France*
[3] *Shenzhen Institute for Quantum Science and Engineering, Southern University of Science and Technology, 518055 Shenzhen, China*
[4] *Faculté des Sciences et Ingénierie, Sorbonne Université, UFR 933, 75005 Paris, France*
[5] *Fakultät für Physik, Ludwig-Maximilians-Universität, Schellingstraße 4, 80799 München, Germany*
[6] *Karlsruher Institut für Technologie, Physikalisches Institut, Wolfgang-Gaede-Str. 1, 76131 Karlsruhe, Germany*
[7] *Karlsruhe Insitute for Technology, Institute for Quantum Materials and Technologies (IQMT), Hermann-von-Helmholtz-Platz 1, 76344 Eggenstein-Leopoldshafen, Germany*
[8] *ICREA-Institució Catalana de Recerca i Estudis Avaçats, 08015 Barcelona, Spain*
[9] *e-mail: hugues.deriedmatten@icfo.eu*
[10] *These authors contributed equally*

Here we discuss aspects relevant to the experiment which have not been addressed in the main text. First, in Supplementary Note 1, we present more details of the optical cavity and the optical setup. Then, in Supplementary Note 2, we describe the technique we use to localize the nanoparticles and to ensure maximal coupling to the cavity electric field. We calculate the size and estimate the number of erbium ions present in the nanoparticle. In Supplementary Note 3 we introduce our cavity stabilization mechanism and estimate the cavity stability. Then, in Supplementary Note 4 we describe the method we derived to switch the cavity resonance at will, characterize the speed of the switch and finally discuss limitations and possible improvements to decrease switching time.

In Supplementary Note 5 we estimate the effect of the off-resonance cavity while measuring the natural lifetime of the emitters. In Supplementary Note 6 we introduce the model used to estimate the multi-exponential decay in the cavity, following a dynamic and a static approach. We compare the data with the model and show that the data is consistent with having a strong Purcell enhancement. Finally, in Supplementary Note 7 we specify the efficiencies of our setup and discuss the feasibility of our current apparatus to detect a single erbium ion and possible modifications to reach high fidelity detection.

SUPPLEMENTARY NOTE 1: CAVITY AND OPTICAL SETUP

The microcavity is composed of a fiber with a concave structure on the tip on which a reflective coating is fabricated. The other side of the cavity is a planar mirror with the same reflective coating as the fiber. To ensure maximum coupling between the ions and the cavity electric field, we add a SiO_2 layer of 245 nm thickness. The thickness of the layer is chosen such that the maximum of the electric field is 40 nm above the SiO_2-air interface, where nanoparticles are deposited. The transmission of the fiber is $\mathcal{T}_f = 100$ parts-per-million (ppm), while for the mirrors $\mathcal{T}_m \approx 2 \times \mathcal{T}_f$ due the presence of the SiO_2 spacer thus leading to a maximum finesse of $F_{\max} \approx 20,000$. The radius of curvature of the concave structure is $r_{oc} \approx 50$ μm and the depth of the structure is close to $p_d \approx 1.5$ μm

The optical setup is shown in Fig 1c. It allows us to perform resonance excitation and detection both via the optical fiber. The 1535 nm laser is used to excite the ions and the 790 nm laser to stabilize the length of the cavity. An acousto-optic modulator (AOM) in a double pass configuration is used to 'pulse' the excitation laser. A single pass AOM operates as excitation/detection 'router'. During excitation, the router AOM is off and the excitation light is directed to the cavity while most of the reflected light is directed back to the same channel. During detection, the router AOM is on and the spontaneous emission from the ions is deflected towards an InGaAs single photon counter (detection efficiency 10 %). A 'filter' double pass AOM after the router is used to add additional protection (60 dB) to the single photon detector during excitation. A wavelength-division multiplexing (WDM) and dichroic mirrors (DM) both for 780/1535 are used for merging the light directed onto the cavity and to separate the transmitted light. The transmitted light is then directed to continuous APDs (PD) for cavity length stabilization and transmission monitor.

SUPPLEMENTARY NOTE 2: LOCALIZING NANOPARTICLES

To localize a nanoparticle, we use scattering loss spectroscopy [1]. While scanning the fiber cavity, we monitor

the cavity transmission which is given by

$$T_c = \frac{4\mathcal{T}_f\mathcal{T}_m}{(\mathcal{T}_m + \mathcal{T}_f + 2B)^2} \tag{SM.1}$$

where $\mathcal{T}_f$ and $\mathcal{T}_m$ are the transmission losses per pass of the fiber and the mirror, $B = 4\sigma/\pi w_0^2$ are the additional losses per pass due to scattering introduced by the nanoparticle with w_0 the cavity mode waist and σ the scattering cross section. The latter can be calculated as

$$\sigma = \left(\frac{2\pi}{\lambda}\right)^4 \frac{\alpha^2}{6\pi} \tag{SM.2}$$

where the polarizability of the nanoparticle is given by [2]

$$\alpha = 3\epsilon_0 V \frac{n^2 - n_{air}^2}{n^2 + 2n_{air}^2} \tag{SM.3}$$

with n and n_{air} the refractive indices of the nanoparticle and surrounding medium, ϵ_0 the vacuum dielectric constant, and $V = 4/3\pi r^3$ the volume of the nanoparticle.

We look for nanoparticles which are big enough to introduce a visible scattering signal, but small enough to maintain the out-coupling efficiency

$$\eta_{out} = \frac{\mathcal{T}_{out}}{(\mathcal{T}_f + \mathcal{T}_m + 2B)} \tag{SM.4}$$

at a high level, where $\mathcal{T}_{out}$ is the out-coupling channel, that is, $\mathcal{T}_f$ or $\mathcal{T}_m$. Fig. 1b (main text) shows a map of the losses that the studied nanoparticle introduces to the cavity at 1535 nm. For $\mathcal{T}_f = \mathcal{T}_m/2 = 100$ ppm, a peak loss $B \approx 43(2)$ ppm is measured when the nanoparticle is well aligned to the cavity mode. For results presented here, the effective cavity length is 6 μm, which includes the field penetration depth of $\sim$ 3 μm inside the refractive coating and the concave structure depth of $\sim$ 1.5 μm. For a radius of curvature of the structure of the fiber of 50 μm, we calculate the beam waist $w_0 = 2.9$ μm. With $n = 1.9317$ the refractive index of Y_2O_3, we infer a nanoparticle radius of 90.5(1.0) nm. Finally, for an erbium doping concentration of 200 ppm, the total number of erbium ions in the crystallographic site of C2 symmetry is close to $11,000$.

SUPPLEMENTARY NOTE 3: CAVITY STABILITY

The cavity is placed on a compact and passively stable nano-positioning platform, which is based on the cryogenic cavity design currently commercialized as qlibri cavity platform, and is robust against the high frequency noise coming from the closed-cycle cryostat. In order to stabilize the cavity length at 1535 nm, which is the ion's resonance, we make use of a second stop band at 790 nm. The coating is designed such that for every 1535 nm mode, there is a close by 790 mode for cavity length stabilization for lengths in the 2 to 20 μm range. Supplementary Figure 1a shows a transmission scan at 1535 nm and 790 nm while scanning the cavity length by close to 1 μm.

To stabilize the length of the cavity, we fine tune the wavelength of the 790 nm laser such that the maximum of the 1535 nm transmission peak overlaps with the middle of the 790 fringe (see Supplementary Figure 1b). Then, the transmission of the red laser is used to monitor cavity drifts and feedback is applied to the piezoelectric crystal to keep this signal at a constant level (side of fringe lock). Supplementary Figure 1c shows the transmission of both lasers as function of the time while the feedback system is on for the whole cryostat cycle, and Supplementary Figure 1e shows a Fast Fourier Transform of the 790 nm transmission signal when the cavity is locked. The dominant noise resides in the $150 - 300$ Hz frequency domain. We note that this is a limiting factor for our cavity stability, as the first eigenfrequency of our setup is close to 1 kHz.

For this particular measurement, the finesse F of cavity at 790 nm is 700 and at 1535 nm is $5,000$. We estimate the cavity stability using both the 790 nm and the 1535 nm transmissions and by modeling the cavity displacement by a single-frequency sinusoidal as $\delta(t) = A\cos(t \cdot 2\pi\nu)$ where ν is set to 200Hz, that is, the main frequency component of the 790 nm transmission signal, and A corresponds to the displacement amplitude. Then, the cavity transmission $T(t)$ as function of time can be calculated as

$$T(t) = T_{\max} \cdot \mathcal{L}(t) = T_{max} \cdot \frac{(\Delta/2)^2}{(\delta(t) + \delta_0)^2 + (\Delta/2)^2} \tag{SM.5}$$

where $\mathcal{L}$ is the Lorentzian spectral line shape of the cavity, δ_0 is the detuning from the center of the line , $T_{\max}$ is maximum of the transmission and $\Delta = \frac{\lambda/2}{F}$ is the full width at half maximum (FWHM).

We then calculate A for both the 790 nm and 1535 nm fringes such that the calculated standard deviation (STD) for long times matches the measured values. Finally, we assess the root mean square (RMS) cavity stability as $\frac{A}{\sqrt{2}}$. For the 790 nm fringe, $\delta_0 = \Delta_{790}/2$, that is, the cavity is stabilized on the side of the fringe at half maximum, and the estimated RMS cavity stability is 31 pm. For the 1535 nm fringe, $\delta_0 = 0$, that is, the cavity is stabilized at the center of the fringe, and the estimated RMS cavity stability is 37 pm. We attribute the discrepancy to a larger uncertainty in the finesse of the 1535 nm mode. The 1535 nm fringes for the finesse measurement were not recorded with high enough resolution, and the obtained value of $5,000$ is therefore a lower bound.

In Supplementary Figure 1c, we can identify time intervals which show significantly smaller signal dispersion, this interval corresponds to the quietest part of the cryostat cycle, which is close to 500 ms. Supplementary Figure 1d corresponds to a zoom in of 30 milliseconds over which the cavity stability is the highest, for which we es-

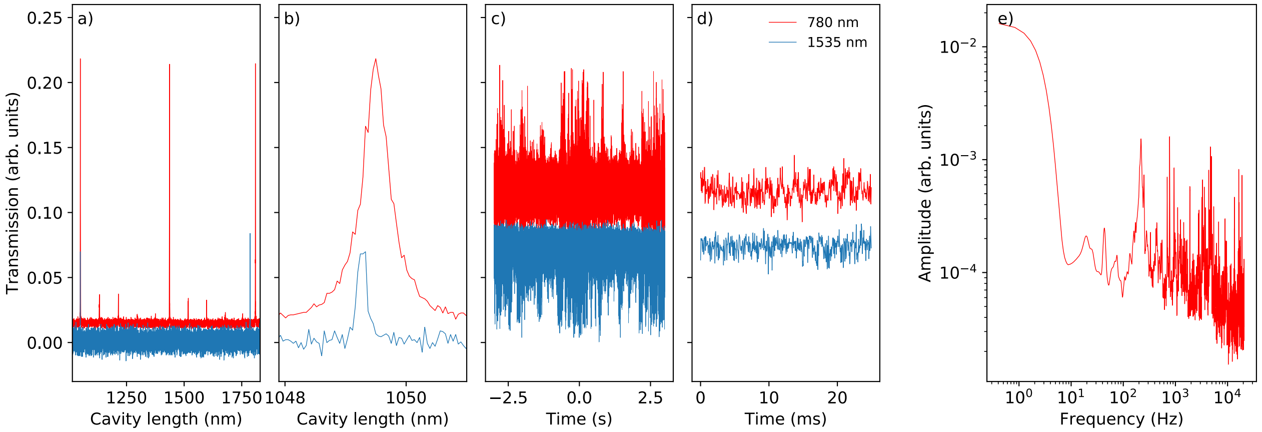

Supplementary Figure 1: Cavity stability measurements. a) 780 nm (red) and 1535 nm (blue) cavity transmission as function of cavity length. For this measurement, the cavity finesse at 780 nm and 1535 nm was 700 and 5,000 respectively. b) Zoom in of (a) on the fringe used to stabilize the cavity length. c) Transmission during 5 seconds while the cryostat is on. The cavity length is actively stabilized to the side fringe of the 780 nm transmission while it is on resonance at 1535 nm. By calculating the standard deviation of the 790 nm transmission, we infer a cavity stability of 30 pm d) Zoom in of (c) on 30 milliseconds over which the cavity stability is the highest. e) Fast Fourier transform of a typical 790 nm transmission signal when the cavity is lock. The dominant noise resides in the $150 - 300$ Hz frequency domain.

timated RMS cavity stability of 21 pm and 31 pm when calculated using the 780 nm and the 1535 fringes respectively.

As seen in this section, our positioner is stable enough to keep the cavity on resonance with the ions during the whole cycle. We note that the cavity stability for the data shown in the main text and in the next sections was slightly degraded, and typical values between 50 and 75 pm RMS were extracted based on the STD of the 1535 nm transmission signal. In the following sections and to calculate the median Purcell factor, for the model and for the cavity switching time we will assume a cavity stability of 62.5 pm RMS, consistent with most of our measurements. The reason for that is not a fundamental limitation. The high stability reported in this section was recorded in the first assembly of the positioner, while for the data shown in the next sections the positioner was re assembled several times. A possibility exists that during the subsequent assemblies a component was not optimally placed resulting in a degraded stability.

SUPPLEMENTARY NOTE 4: CAVITY RESONANCE SWITCHING

As discussed in the main text, we switch the cavity resonance by a fast change of a voltage offset on a piezoelectric and by stabilizing the cavity to either side of the 790 nm cavity fringe at will. Between the two sides of the fringe, the total cavity length displacement is $\Delta_{790} = \text{FWHM}_{790}$. The 1535 nm resonance with linewidth Δ overlaps with the middle of one side of the 790 nm fringe (see Fig. 3a and description from main text), thus when the cavity is stabilized to the opposite side, erbium ions are detuned from the cavity.

Now, we characterize the reduction of the Purcell factor and the spontaneous emission rate in the cavity mode during the time the cavity resonance is switched. The Purcell factor as function of the detuning δ from the cavity resonance Δ is then given by

$$C(\delta) = \mathcal{L}(\delta) \cdot C, \tag{SM.6}$$

where

$$\mathcal{L}(\delta) = \frac{(\Delta/2)^2}{\delta^2 + (\Delta/2)^2}, \tag{SM.7}$$

is the normalized Lorentzian spectral line of the cavity.

The detected countrate as function of the time is given by [3]

$$r(t) = \frac{C(t)}{\tau_n} \cdot \mathcal{N}(t) \tag{SM.8}$$

where τ_n is the natural lifetime, $\mathcal{N}(t)$ is the number of ions in the excited state and $C(t)$ is the Purcell factor. The number of ions in the excited state can be calculated as

$$\mathcal{N}(t) = \mathcal{N}_0 e^{-\frac{t}{\tau_n} - \int_0^t \frac{C(t')}{\tau_n} dt'} \tag{SM.9}$$

where $\mathcal{N}_0$ is the number of ions in the excited state before the cavity frequency is shifted. The first term in the exponent of Eq. SM.9 takes into account the decrease in

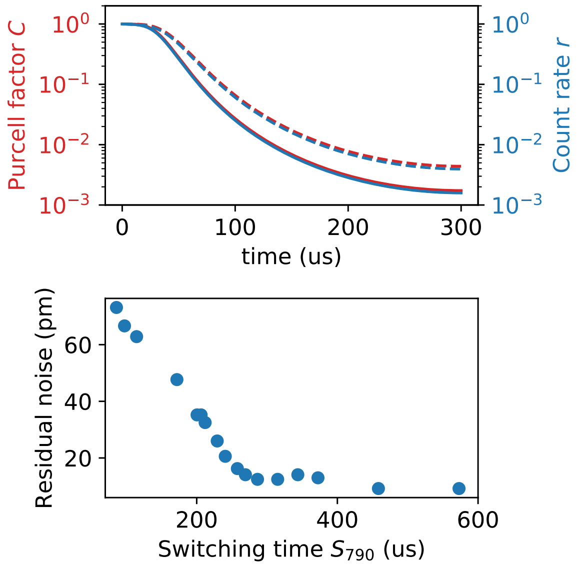

Supplementary Figure 2: a) Reduction of the Purcell factor (red) and the count-rate (blue) as function of time during $S_{790} = 300$ μs. Solid lines is for the ideal detuning of $\delta = \frac{\Delta_{790}}{\Delta_{1535}}\Delta = 12\Delta$ and dashed line for $\delta = \frac{\Delta_{790}}{\Delta_{1535,eff}}\Delta = 7.5\Delta$, the value obtained considering the cavity stability. b) Residual noise as function of the switching time S_{790}

population due to emission in free space, while the second term does it in the cavity mode. Eq. SM.9 only takes into account the modification in the density of states and neglects light-matter dynamics [3].

In order to minimize coupling of mechanical noise in the cavity, the detuning as function of time, that is, the voltage applied to the piezoelectric transducer, is given by

$$\delta(t) = \Delta_{790} \cdot \sin^2\left(\frac{\pi t}{2S_{790}}\right) \qquad \text{(SM.10)}$$

where S_{790} is the switching time between the two locking points.

For this experiment, we use $S_{790} = 300$ μs. Assuming the cavity resonance at 1535 nm is $\Delta_{1535} = \Delta_{790}/12$ and a Purcell factor 15, we expect a total reduction of 1/577 in the Purcell factor and 1/630 in the countrate (see Supplementary Figure 2a). Depending on the practical purpose a faster decoupling time might be required, which can be achieved at the expenses of a smaller reduction of the Purcell factor or the countrate. Here we define the switching time S_{1535} as the time needed to decrease the countrate by a factor of 10. For ideal parameters, we therefore expect $S_{1535} = 67$ μs.

Now, we compare the expected reduction of the Purcell factor and the countrate with the measured values. Fig. 3e and 3f in the main text show the detected countrate as a function of time at the moment the cavity is tuned off- and on-resonance. The solid line is the model defined in Eq. SM.8. To take into account the cavity stability, we introduce an effective cavity linewidth defined as

$$\Delta_{1535,eff} = \sqrt{\Delta_{1535}^2 + \text{Noise}_{\text{RMS}}^2} \qquad \text{(SM.11)}$$

with $\text{Noise}_{\text{RMS}} = 62.5$ pm RMS as measured (see Supplementary Note 3).

For these parameters, the maximum reduction of the Purcell factor is 1/227 and for the countrate is 1/253.

In Fig. 3e, the first vertical line indicates the beginning of the switching and the second is the time at which the countrate is reduced by a factor of 10, giving a $S_{1535} = 85(15)$ μs. In Fig. 3f, the second vertical line indicates the end of the switching and the first is the time at which the detected countrate is a factor of 10 smaller than the maximum, for which the time interval is 87(16) μs. Here, we see that by the time the cavity is back on resonance, the maximum countrate cannot be reached as population is lost during the switching process which occurs in a time scale comparable to the Purcell enhanced decay time.

In order to reduce further the switching time (assuming the cavity stability is not the limiting factor), one can simply move the piezo faster thus reducing S_{790}. However, a faster kick leads to increased residual mechanical noise. Supplementary Figure 2b shows the residual noise as function of the switching time S_{790}. The residual noise is calculated as the standard deviation of the locking signal in the first 1 ms after the cavity is set on resonance at 1535 nm. As expected, the residual noise increases for a faster switching time S_{790}, that is, for faster switching frequency. We estimate the switching frequency as $1/(2 \times S_{790}) \approx 1.6$ kHz. The characteristic frequency of the residual noise is between $7 - 10$ kHz. We attribute this residual motion to the fiber in the plane perpendicular to the cavity axis. To first order, the cavity length should be robust against the lateral displacement of the fiber. However, a misalignment when placing the fiber in the positioner could lead to strong coupling between them, as confirmed in our experiment. We see that while moving the fiber laterally, the cavity length is shifted, and we estimate the coupling to be in the $20 - 40\%$ range.

In order to increase the switching time S_{1535}, we can follow several strategies:

- perform a proper alignment of the fiber in order to minimize the coupling between the vibration modes,
- increase the frequency of all mechanical eigenmodes,
- increase the finesse of the 1535 nm resonance,
- use a faster growing function than $\sin^2$ or implement iterative learning algorithms to shape the signal sent to the piezo to minimize added noise

Altogether, we estimate that values of S_{1535} in the microsecond scale could be achievable by combing these improvements if the cavity stability issue is addressed.

SUPPLEMENTARY NOTE 5: EMITTERS COUPLED TO AN OFF-RESONANCE CAVITY

In Fig. 3c and the following text in the manuscript, we show how the cavity resonance switching can be used to extract the natural lifetime of a single or an ensemble of emitters. For that, we set the cavity on resonance with the ions and we excite them. Immediately after, we tune the cavity off resonance. After a time t_d (see main text for definition), we tune back the cavity on resonance and a strong fluorescence is recorded (Fig. 3c main text). By plotting the counts in a given time window as function of t_d, one can extract the natural lifetime (Fig. 2a, green crosses main text). However, one needs to consider the influence of the off-resonance cavity, which under certain conditions could lead to an inhibited emission and increased lifetime.

In earlier work [4] we have performed FDTD simulations on the emission lifetime of a dipole placed on a planar Bragg mirror and found that this reduces the excited state lifetime by up to a factor 1.3. This means that already the presence of a single mirror leads to some Purcell-enhanced emission rate. This prediction is in agreement with fluorescence decay time measurements performed on a bunch of nanoparticle in free space, for which we extract a natural lifetime of 13.94(1) ms (see Supplementary Figure 3). This value is exactly 1.3 times larger than the 10.8(3) ms measured for the studied nanoparticle which is placed on the Bragg mirror. Notably, the effect remains smaller than the analytical prediction for a perfect mirror, where a reduction by up to a factor 2 is expected. This is related to the finite angular range where the Bragg mirror is reflective, and due to the coupling into Bloch modes.

For an off-resonant cavity, the same reasoning remains valid, and emission suppression will be due to destructive interference of partial waves within a narrow solid angle only. The specific geometry of the mirror defines the quantitative value of the suppression, in particular the geometry of the concave profile. In our case, this is a very shallow profile (structure depth $\sim 1.5\ \mu$m, radii of curvature of 50 μm), such that only modes within a small angular range of $\alpha \approx 6\,$deg will be suppressed. A comparable geometry of an open access microcavity, however with larger aspect ratio and thus larger angular range ($\alpha \approx 22$) was studied in [5], where a suppression of 32% was calculated by FDTD. This fits reasonably well to the fraction of the 4π-solid angle that the geometry covers, where $\Omega = 2 \times 2\pi(1 - \cos(\alpha))$ is the estimated effective solid angle that is relevant for the suppression. For $\alpha = 22\,$deg, this yields a 29% suppression, close to the FDTD result. Translated to our geometry, for $\alpha = 6\,$deg, we obtain a negligible lifetime suppression of $< 3\%$.

As a second indicator that the effect should be small in our setup, we refer to our earlier work with NV centers coupled to a planar Fabry-Perot mode (see [6]). Only for very small mirror separations we were able to observe notable lifetime increase for an off resonant cavity (Fig. 4a in [6]).

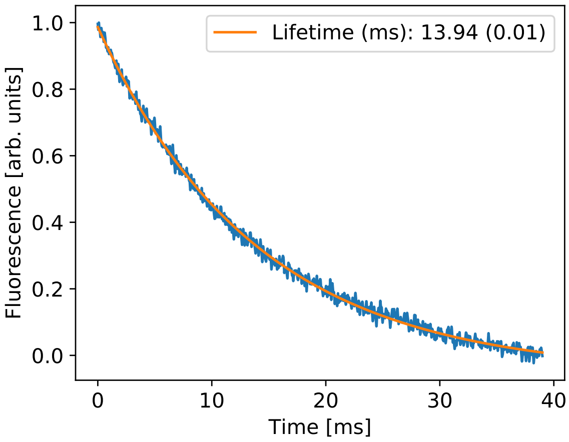

Supplementary Figure 3: Excited state lifetime of erbium doped nanoparticles measured in free space.

To summarize, in the data shown in Fig. 2a in the main text in order to extract the natural lifetime, we estimate that the effect of the off-resonance cavity is negligible, but can be of relevance for different geometries. We note that by performing measurements with dynamic switching at different mirror separations, it will be possible to quantify the effect of emission suppression, such that the measurement principle indeed allows one to obtain an accurate value of the Purcell factor. We note however, that the presence of the mirror carrying the sample comes in addition, and needs to be quantified separately. Comparing to a crystal in free space would thus yield larger values of the Purcell factor.

SUPPLEMENTARY NOTE 6: MODEL FOR PURCELL ENHANCED DECAYS OF AN ENSEMBLE OF SOLID-STATE EMITTERS IN A CAVITY

As discussed in the main text, the recorded signal in order to extract the Purcell enhanced lifetime shows a strong indication of multi-exponential behavior. The reason for that is that ions with different Purcell enhancement contribute to the detected signal. In this section we derive a model that considers a large ensemble of ions in a nanoparticle that experience a maximum Purcell factor $C_{\max}$ which is then reduced due to three main factors. The first factor is related to the orientation of the dipole moments of the ions. To estimate this first contribution, we consider a spherical nanoparticle of radius r, which is composed from several crystalline structures. Each crystalline structure supports three orthogonal directions for the orientation of the dipoles. Already for a small number of structures, it is reasonable to assume that the dipole orientations are random and homogeneously distributed. For a cavity axis along $\hat{z}$ and considering that both com-

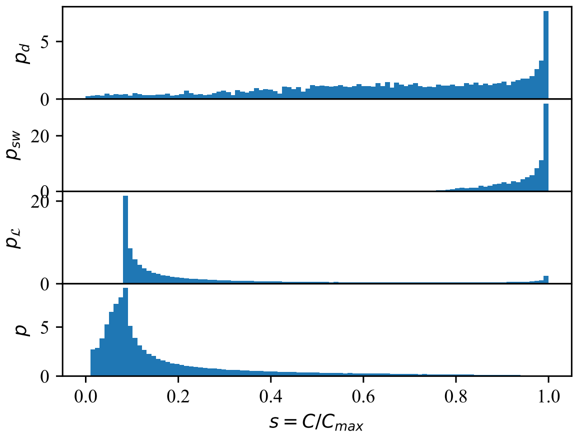

Supplementary Figure 4: Normalized distributions p_{d} and p_{sw} obtained after making histograms of $R_{i,d}$ and $R_{i,sw}$ over a large number of randomly placed ions with randomly oriented dipoles. The distribution $p_{\mathcal{L}}$ is calculated following a stroboscopic approach on the values of $R_{\mathcal{L}(t,\delta_0)}$ over a large sampling space. Finally, the normalized distribution p is calculated from p_{d}, p_{sw} and $p_{\mathcal{L}}$ as described in the text.

ponents of the cavity electric field $\bar{E} = E_x\hat{x} + E_y\hat{y}$ have the same resonance frequency, each ion i in the nanoparticle experiences a reduced Purcell $C_{\max} \cdot R_{i,\mathrm{d}}$ with

$$R_{i,\mathrm{d}} = (\hat{x}\cdot\hat{n}_i)^2 + (\hat{y}\cdot\hat{n}_i)^2 = n_{i,x}^2 + n_{i,y}^2 \qquad \text{(SM.12)}$$

where $n_{i,\{x,y\}}$ are the components of the dipole moment $\bar{d}_i = d_i\hat{n}_i$ along the x and y axes.

Next, we consider the finite extension of the nanoparticle with respect to the standing wave of the cavity field, meaning that ions far from the center experience an additional reduction of the Purcell factor. This reduction is given by

$$R_{i,\mathrm{sw}} = \cos\left(\frac{z_i + z_{\mathrm{offset}}}{\lambda}\cdot 2\pi\right)^2 \qquad \text{(SM.13)}$$

where z_i is the distance of the ion i in the z-axis from the center of crystal, and z_{offset} is the distance of the center of the crystal to the maximum of the standing wave.

Finally, we model the fluctuation of the cavity by a single-frequency sinusoidal as $\delta(t) = A\cos(t\cdot 2\pi\nu)$ where ν is the main frequency component of the 790 nm transmission signal, and A corresponds to the cavity displacement amplitude (see section 'Cavity stability'). This affects equally all ions, thus the Purcell factor is globally reduced by

$$R_{\mathcal{L}(t,\phi)} = \frac{(\Delta/2)^2}{(\delta(t)+\delta_0)^2 + (\Delta/2)^2} \qquad \text{(SM.14)}$$

where $\mathcal{L}$ is the Lorentzian spectral line shape of the cavity, $\Delta = \frac{\lambda/2}{F}$ is the FWHM and δ_0 is detuning of the cavity at the time a particular trial of the experiment starts.

Now, we model the detected counts as function of time following two different strategies: a dynamic one and a static one. For the dynamic approach, we assume a large number of ions and calculate the probability to detect photons as function of time which is mainly determined by the time-dependent Purcell factor. For the static one, we reconstruct the detected counts by calculating the distribution of decays rates present in our system. The main difference between these two methods is that the frequency at which the cavity vibrates is relevant only for the first method (see discussion below).

To model the counts per bin as function of the time following the dynamic strategy, we consider a large ensemble of ions which are excited and start to decay. The probability that an ion i emits in the cavity as a function of time is proportional to

$$e^{-\int_0^t \frac{C(t',i,\delta_0)}{\tau_n}dt'}, \qquad \text{(SM.15)}$$

with a time-dependent Purcell factor given by

$$C(t,i,\delta_0) = C_{\max}\cdot R_{i,\mathrm{d}}\cdot R_{i,\mathrm{sw}}\cdot R_{\mathcal{L}(t,\delta_0)}. \qquad \text{(SM.16)}$$

The total detected counts as function of the time is estimated by averaging over a large number of ions with randomly chosen initial cavity detunings δ_0, that is,

$$\mathrm{cts}(t) \approx \eta \sum_i^{\#i}\sum_{\delta_0}^{\#\delta_0} \frac{e^{-\int_0^t \frac{C(t',i,\delta_0)}{\tau_n}dt'}}{\#i\,\#\delta_0} \qquad \text{(SM.17)}$$

with $\#i$ and $\#\delta_0$ the number of ions and detunings which are considered, and η is a normalization factor that includes the detection efficiency and the number of ions which are excited at the beginning of the experiment.

For the static approach, we model the detected counts as linear superposition of exponential decaying curves, that is,

$$\mathrm{cts}(t) \approx \int_0^1 p(s)e^{-\frac{s\,t\,C_{\max}}{\tau_n}}ds. \qquad \text{(SM.18)}$$

with $s = C/C_{\max}$ and $p(s)$ the normalized distribution of Purcell factor present in the system. We start by discretizing eq. SM.18 such that

$$\mathrm{cts}(t) \approx \sum_{j=1}^{n} p(s_j)e^{-\frac{s_j\,t\,C_{\max}}{\tau_n}}\Delta_s \qquad \text{(SM.19)}$$

with $s_j = [1/n, 2/n, ..., 1]$, n the number of discrete steps and $\Delta_s = 1/n$ is the step size. To estimate $p(s_j)$, we first calculate the discrete distributions $p_{\mathrm{d}}(s_d)$, $p_{\mathrm{sw}}(s_{sw})$ and $p_{\mathcal{L}}(s_{\mathcal{L}})$ corresponding to the distributions of the dipole orientations of the ions, the position of the nanoparticle with respect to the standing wave and the fluctuations of the cavity respectively. Note $s_d, s_{sw}, s_{\mathcal{L}}, = [1/n, 2/n, ..., 1]$. $p_{\mathrm{d}}(s_d)$ and $p_{\mathrm{sw}}(s_{sw})$ are calculated by

making normalized histograms of $R_{i,\mathrm{d}}$ and $R_{i,\mathrm{sw}}$ by placing ions randomly and homogeneously distributed in the nanoparticle with randomly oriented dipole moments. Note that the bin size of the histogram is set to be the same as the step size $1/n$ of the discrete distribution, such that the bins correspond to [(0-1/n), (1/n-2/n), ..., -1)].

The distribution $p_{\mathcal{L}}$ cannot be directly calculated because it is a time dependent function. We therefore estimate it following a stroboscopy approach. That is, $p_{\mathcal{L}}$ is given by the normalized histogram of $R_{\mathcal{L}(t,\delta_0)}$ calculated at random times t in a time interval larger than the chosen period of the cavity vibration.

We now have three lists, which correspond to the three discrete distributions, of the form (s, p) with n elements each. $p(s_j)$ is then calculated as follows: first, we make a new list (s', p') containing n^3 elements where

$$s' = s_d[k] \cdot s_{sw}[l] \cdot s_{\mathcal{L}}[m], \qquad \text{(SM.20)}$$

$$p' = p_{\mathrm{d}}(s_d)[k] \cdot p_{\mathrm{sw}}(s_{sw})[l] \cdot p_{\mathcal{L}}(s_{\mathcal{L}})[m], \qquad \text{(SM.21)}$$

where $k, l, m = [1, 2, ..., n]$ are indices for elements of the corresponding lists. We then construct a new histogram out of the list (s', p') with bin size set to $1/n$. Finally, the histogram is normalized to obtain $p(s_i)$. The obtained distributions p_{d}, p_{sw}, $p_{\mathcal{L}}$ and p are shown in Supplementary Figure 4.

The main difference between the static and the dynamic methods arises mainly in how $p_{\mathcal{L}}$ is calculated. In our calculation, the frequency of the cavity vibration ν does not influence the value of $p_{\mathcal{L}}$. We now illustrate how this small difference strongly affects the model. First, we consider the case where the time scale of the cavity vibration $1/\nu$ is much slower than the slower lifetime τ' present in the system, that that is, $1/\nu \ll \tau'$. In this case, in some trials of the experiment, we will record very fast photons as the cavity is on resonance for a time longer than $\tau_{C_{\max}}$, while moments later, when the cavity is slowly moving out of resonance, slower photons will be recorded. After integrating counts for a very long time, fast and slow contributions should be clearly visible in the data. Now, we consider the opposite case in where $1/\nu \gg \tau'$, that is, very fast cavity vibration compared to the slower decay rate present in the system. In this case, on each trial, we can assume that the cavity will be on average enough time on resonance such that all the ions had already decayed at early times of the trial, thus no photons will be recorded at later stages. In this case, for long integration times, a slow component is not expected in the signal, although the cavity displacement can be assumed to be the same as in the opposite case. The influence of the frequency of the vibrations of the cavity on the ions can only be studied by the dynamic model and not by the static one.

Fig. 2a-c in the main text shows both the dynamic and the static model for the parameters of our system. The radius of the particular is set to 90 nm consistent with measurements. The finesse of the cavity is 16'000 for which $\Delta = 50$ pm. The offset of the maximum of the standing wave with respect of the crystal z_{offset} is 50 nm

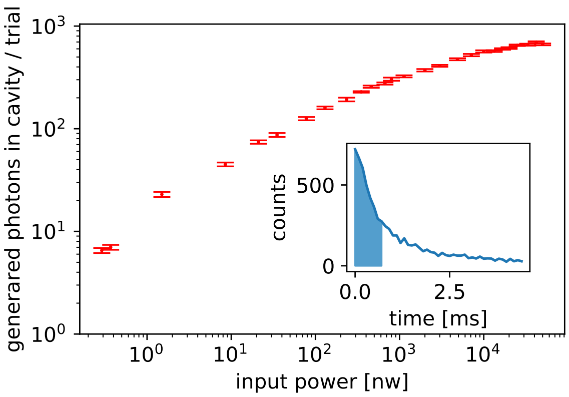

Supplementary Figure 5: Number of photons generated in the cavity mode as a function of the excitation power before the cavity. Excitation pulse length is 500 μs. Error bars represent one standard deviation of photons counts. The inset shows the detection window $[0 - \tau_c]$, with $\tau_c = 0.7$ μs.

(see Supplementary Note 1). The amplitude of the cavity fluctuations is set to $A = \mathrm{Noise}_{\mathrm{RMS}} \cdot \sqrt{2} = 88.4$ pm where $\mathrm{Noise}_{\mathrm{RMS}} = 62.5$ pm and the frequency of the cavity displacement $\nu = 200$ Hz (see Supplementary Note 3) . The expected maximum Purcell factor of $C_{\max} = 171$ fails to describe the data well, but an almost perfect overlap is obtained by choosing $C_{\max} = 150$. Finally, the remaining parameter is the normalization factor. The normalization factor is related to the number of ions that are excited after the pulse and the detection efficiency. This factor is difficult to assess as the number of excited ions strongly depends on the power broadening which depends on the homogeneous linewidth which is unknown. The normalization is set by minimizing the RMSE between the model and the data set. Finally, on each bin we add counts to account for the measured dark count rate of 13.7 Hz. As seen in the Fig. 2a in the main text, both models stay consistently below one standart deviation from the data. The dynamic model fails to describe the slower components, thus suggesting that slower component in the cavity vibration are present. These slower components can be explained by the 2 Hz cycle of the cryostat.

In conclusion: we presented a model that accurately describes the data. From the model we conclude that the early fast decay seen in the data can only be explained if a small ensemble of ions experience a large Purcell factor which is then reduced. The main reduction is related to the cavity stability. In case the cavity stability improves, ions in the center of the nanoparticle and with the dipole moment aligned to the cavity electric field could be in principle isolated yielding to an up to five times higher signal to noise ratio, a level at which single ion detection would be feasible in our setup (see Supplementary Note 7).

SUPPLEMENTARY NOTE 7: NUMBER OF DETECTED IONS

The probability p_{det} to detect a photon generated in the cavity mode is given by

$$p_{det} = \eta_{out} \times \eta_{mm} \times \eta_{col} \times \eta_{det} \times \eta_g \quad \text{(SM.22)}$$

where η_{out} is the probability of the photon leaving through the fiber as defined in Eq. SM.4, η_{mm} is the mode matching between the fiber and the cavity mode [7], η_{col} is the collection path efficiency, η_{det} is the detector efficiency and η_g is the proportion of the single photon in the detection time window.

For our cavity, $\eta_{out} = 0.25$ when detecting via fiber and when the particle is aligned to the cavity mode, η_{mm} is calculated to be 0.60, η_{col} is measured to be 0.3, $\eta_{det} = 0.1$, and $\eta_g = 0.63$ for a detection time window $t_{det} = [0 - \tau_c]$ where τ_c is the lifetime of the emitter under Purcell enhancement. All together then results in $p_{det} = 0.28\%$. Supplementary Figure 5 shows a measurement of the number of photons generated in the cavity as a function of the excitation input power at the input of the cavity. For an input power of 7 nW as in Fig. 2 (main text), the detected signal corresponds to 40 intra cavity photons generated by 80 ions (an ion is excited with almost 50% probability, see Eq. SM.25). For the first data point of Supplementary Figure 5 with input power of 330 pW, the detected signal corresponds to 5 intra cavity photons generated by 10 ions.

In order to detect a single ion, we need to compare the probability of detecting a photon p_{det} with the probability of detecting noise in the same time window t_{det}. We define the signal to noise ratio as

$$S/N = \frac{p_{det}}{p_n}, \quad \text{(SM.23)}$$

where p_n is the background probability in the detection window t_{det}, in our case mostly due to the dark-count rate of the single photon detector (10 Hz). We then calculate

$$S/N = \frac{p_{det}}{\tau_c} \times \frac{1}{10 \text{ Hz}} = 0.5. \quad \text{(SM.24)}$$

This means that the number of photons that must be generated in the cavity to achieve a $S/N = 1$ is equal 2. The probability p_{gen} for an ion to emit a single photon in the cavity is indeed given by

$$p_{gen} = p_{exc} \times \chi_{cav} \times \beta \approx 0.47, \quad \text{(SM.25)}$$

where p_{exc} is the excitation probability (0.5 since we excite incoherently). We therefore need to detect the fluorescence from four ions to achieve a $S/N = 1$, which is currently not sufficient to reach high-fidelity detection of a single ion.

Several solutions can be implemented to increase the signal in order to detect a single ion. First, with the use of a superconducting nanowire single photon detector, which are specified to have detection efficiencies of close to 80% (efficiency of our current detector is 10%). With this detector, the sensitivity could be increased by at least a factor of 8, thus single photon detection would be already possible with no extra modification to the setup. In case the cavity stability is improved, ions that emit photons at a rate which is up to five times faster than the average can be in principle addressed, leading to an additional factor of 5 in the signal to noise ratio. In addition, in our current cavity most of the light is emitted on the planar mirror side ($\mathcal{T}_m = 2\mathcal{T}_f$). Future cavities with higher reflective mirrors will significantly increase the escape efficiency through the fiber. Even more, new mirrors will also lead to significantly higher cavity finesse and therefore increased Purcell enhancement. By increasing the reflectivity of the mirror in order to have a finesse of 40,000, we expect that the cavity improvements will lead to a detection sensitivity improved by a factor 4. We can therefore expect an increase in detection sensitivity by a factor 120 compared to the current sensitivity, which should allow us to reach high fidelity single photon detection.

SUPPLEMENTARY REFERENCES

[1] M. Mader, J. Reichel, T. W. Hänsch, and D. Hunger, A scanning cavity microscope, Nature communications **6**, 7249 (2015).

[2] M. Wind, J. Vlieger, and D. Bedeaux, The polarizability of a truncated sphere on a substrate i, Physica A: Statistical Mechanics and its Applications **141**, 33 (1987).

[3] H. Thyrrestrup, A. Hartsuiker, J.-M. Gérard, and W. L. Vos, Non-exponential spontaneous emission dynamics for emitters in a time-dependent optical cavity, Optics express **21**, 23130 (2013).

[4] B. Casabone, J. Benedikter, T. Hümmer, F. Oehl, K. de Oliveira Lima, T. W. Hänsch, A. Ferrier, P. Goldner, H. de Riedmatten, and D. Hunger, Cavity-enhanced spectroscopy of a few-ion ensemble in eu3+: Y2o3, New Journal of Physics **20**, 095006 (2018).

[5] Z. Di, H. V. Jones, P. R. Dolan, S. M. Fairclough, M. B. Wincott, J. Fill, G. M. Hughes, and J. M. Smith, Controlling the emission from semiconductor quantum dots using ultra-small tunable optical microcavities, New Journal of Physics **14**, 103048 (2012).

[6] H. Kaupp, T. Hümmer, M. Mader, B. Schlederer, J. Benedikter, P. Haeusser, H.-C. Chang, H. Fedder, T. W. Hänsch, and D. Hunger, Purcell-enhanced single-photon emission from nitrogen-vacancy centers coupled to a tunable microcavity, Physical Review Applied **6**, 054010 (2016).

[7] D. Hunger, T. Steinmetz, Y. Colombe, C. Deutsch, T. W.

Hänsch, and J. Reichel, A fiber fabry–perot cavity with high finesse, New Journal of Physics **12**, 065038 (2010).
